# Preparation of Peptide and Recombinant Tissue Plasminogen Activator Conjugated Poly(Lactic-Co-Glycolic Acid) (PLGA) Magnetic Nanoparticles for Dual Targeted Thrombolytic Therapy

**DOI:** 10.3390/ijms21082690

**Published:** 2020-04-13

**Authors:** Huai-An Chen, Yunn-Hwa Ma, Tzu-Yuan Hsu, Jyh-Ping Chen

**Affiliations:** 1 Department of Chemical and Materials and Materials Engineering, Chang Gung University, Kwei-San, Taoyuan 33302, Taiwan; eam012331@gmail.com; 2 Department of Physiology and Pharmacology and Healthy Aging Research Center, Chang Gung University, Kwei-San, Taoyuan 33302, Taiwan; yhma@mail.cgu.edu.tw (Y.-H.M.); w1314lin@gmai.com (T.-Y.H.); 3 Department of Plastic and Reconstructive Surgery and Craniofacial Research Center, Chang Gung Memorial Hospital, Linkou, Kwei-San, Taoyuan 33305, Taiwan; 4 Research Center for Food and Cosmetic Safety, Research Center for Chinese Herbal Medicine, College of Human Ecology, Chang Gung University of Science and Technology, Taoyuan 33302, Taiwan; 5 Department of Materials Engineering, Ming Chi University of Technology, Tai-Shan, New Taipei City 24301, Taiwan

**Keywords:** magnetic nanoparticles, tissue plasminogen activator, poly(lactic-co-glycolic acid), targeted drug delivery, clot lysis, nanomedicine

## Abstract

Recombinant tissue plasminogen activator (rtPA) is the only thrombolytic agent that has been approved by the FDA for treatment of ischemic stroke. However, a high dose intravenous infusion is required to maintain effective drug concentration, owing to the short half-life of the thrombolytic drug, whereas a momentous limitation is the risk of bleeding. We envision a dual targeted strategy for rtPA delivery will be feasible to minimize the required dose of rtPA for treatment. For this purpose, rtPA and fibrin-avid peptide were co-immobilized to poly(lactic-co-glycolic acid) (PLGA) magnetic nanoparticles (PMNP) to prepare peptide/rtPA conjugated PMNPs (pPMNP-rtPA). During preparation, PMNP was first surface modified with avidin, which could interact with biotin. This is followed by binding PMNP-avidin with biotin-PEG-rtPA (or biotin-PEG-peptide), which was prepared beforehand by binding rtPA (or peptide) to biotin-PEG-maleimide while using click chemistry between maleimide and the single –SH group in rtPA (or peptide). The physicochemical property characterization indicated the successful preparation of the magnetic nanoparticles with full retention of rtPA fibrinolysis activity, while biological response studies underlined the high biocompatibility of all magnetic nanoparticles from cytotoxicity and hemolysis assays in vitro. The magnetic guidance and fibrin binding effects were also confirmed, which led to a higher thrombolysis rate in vitro using PMNP-rtPA or pPMNP-rtPA when compared to free rtPA after static or dynamic incubation with blood clots. Using pressure-dependent clot lysis model in a flow system, dual targeted pPMNP-rtPA could reduce the clot lysis time for reperfusion by 40% when compared to free rtPA at the same drug dosage. From in vivo targeted thrombolysis in a rat embolic model, pPMNP-rtPA was used at 20% of free rtPA dosage to restore the iliac blood flow in vascular thrombus that was created by injecting a blood clot to the hind limb area.

## 1. Introduction

Within the two ischemic and hemorrhagic categories of stroke, the ischemic stroke accounts for ~87% of all cases, according to the American Stroke Association [1]. A multitude of molecular pathways may be involved in the onset and progression of ischemic stroke, thus an equally diverse arsenal of intervention strategies is needed. To date, the gold standard for intravenous (IV) intervention of ischemic stroke is by administrating recombinant tissue plasminogen activator (rtPA), a thrombolytic drug that dissolves clots to restore blood flow. As a serine protease, rtPA is the major enzyme that is responsible for clot dissolution by catalyzing the conversion of plasminogen to plasmin. It is currently the only thrombolytic agent approved by the U.S. Food and Drug Administration (FDA) for the treatment of ischemic stroke [2]. A major issue in rtPA therapeutic use is the short half-life (4–8 min) in the circulation due to autolysis and the influences of inhibitors, enzymes, and antibodies in blood [3]. The poor affinity of rtPA toward thrombus also reduces its thrombolysis efficacy and might cause ischemia reperfusion injury to the neuron beyond the thrombolysis window [4]. Other limitations of rtPA for clinical use include the short window time for treatment (usually within 3 h) and the risk of hemorrhagic side effects [5]. Consequently, several strategies have emerged to improve the thrombolytic efficacy of rtPA, such as ultrasound-based thrombolysis, targeting thrombolysis, and immobilized thrombolytic drug to improve the safety and effectiveness of thrombolytic therapy [6]. 

Nanomedicine using nanoparticles for drug delivery is a novel field for the diagnosis and treatment of diseases. Although with similar biologic molecular scale when compared with traditional medicine, the unique properties of nanoparticles provide more strategic advantages and application flexibility over pure molecular therapeutics [7]. Using nanoparticles as rtPA carrier for thrombolytic therapy has also been widely explored. For example, biodegradable polymers conjugated with rtPA can provide the protection form the inhibitor in the circulation to prolong its circulation time, which might also confer the possibility for controlled release in encapsulated drug formulation [8]. In recent years, magnetic nanoparticles (MNP), especially iron oxide (Fe_3_O_4_) MNP, are frequently employed as a nano-carrier for drug delivery. Not only to be useful as a magnetic resonance imaging contrast agent, the MNP can also be endowed with other advantages, such as magnetic targeting (physical targeting) and localized heating by magnetic field induction or near-infrared laser irradiation [9]. In addition to pristine nanoparticles, MNP could be also surface modified to enhance their functionality as a drug delivery vehicle. The most common example is surface modification with polyethylene glycol (PEG) to prevent uptake by the mononuclear phagocyte system during circulation, thus prolonging the life time of injected drug [10]. Conjugation with ligand is another commonly used strategy for nanoparticle-based targeted drug delivery. Indeed, ligand-mediated drug delivery could offer a highly specific binding between the ligand molecule immobilized to the nano-carrier and the receptor molecule highly expressed in the diseased area, which could enhance the treatment efficacy by inducing drug accumulation and increasing local drug concentration in the targeted area [11].

Many groups have proposed nanomedicine using targeted rtPA delivery strategy for thrombolysis [12]. We have extensively studied different MNP-based polymeric nano-carrier for magnetically targeted delivery of rtPA, being prepared either by immobilization of rtPA on the particle surface or by encapsulation within the polymeric matrix, and demonstrated the improved thrombolytic efficacy both in vitro and in vivo [13,14,15]. Recently, we also pioneered the use of thermosensitive magnetic liposomes for targeted delivery and temperature-sensitive release of rtPA [16,17]. Other groups used different ligands for targeting fibrin in a blood clot or different moieties that are associated with the blood clot. For example, the ligands used for rtPA delivery targeting thrombus include peptide targeting FXIII [18], fibrin antibody targeting fibrin [19], Arg-Gly-Asp (RGD) [20], cyclicRGD [21], or other peptides [22] targeting GP IIb/IIIa, and fucoidan targeting p-selectin [23]. Those targeted thrombolysis strategies have demonstrated promising results in improving thrombolytic efficiency with shorter clot lysis time, which makes possible the use of reduced drug dosage to circumvent associated side effects, such as the bleeding risk [24].

Consider a ligand targeting fibrin, which is a trimeric molecule consisting of α-, β-, and γ-chains and a major constituent of fresh or old blood clots. Although the actual quantity of fibrin content in a clot varies from clot to clot, fibrin is the major component in a clot and it exists on the surface of a clot that is slowly dissolving either spontaneously or during therapeutic thrombolytic intervention. It is conceivable that a peptide specific for fibrin could be labelled with radioisotopes and used as an imaging agent to detect vascular thrombosis. This was demonstrated from a previous study using 99mTc labelled pentapeptide Gly-Pro-Arg-Pro-Pro (GPRPP), which has high affinity for the fibrin α-chain, for the imaging of vascular thrombosis in animals [25]. The peptide GGSKGC was later added to the C-terminus of GPRPP in order to impart functionality on the peptide. This peptide (GPRPPGGSKGC) was later shown to have high fibrin-avid affinity and high resistance to proteolysis [26]. 

Although using a magnetic field to magnetically guide the movement of MNP for rtPA delivery is promising, the optimization of a magnetic field might be the limiting factor, especially for clinical application. On the other hand, ligand targeting for rtPA delivery might pose challenges, such as specificity and possible immunogenicity of the targeting moiety. Therefore, a dual targeting mechanism for rtPA and for other thrombolytic drugs delivery in general is warranted. This dual targeted strategy for rtPA delivery has rarely been investigated [21]. A recent paper using two peptides as ligands to construct dual targeted liposomes for delivery of a neuroprotective agent proved to be a safe and effective treatment for ischemic stroke [27]. By integrating different targeting functionalities, dual and even multi-targeted nanoparticles could be designed to provide a paradigm for precise drug delivery to the targeted site. Through sequential or synchronized navigation of the drug-loaded nano-sized carrier, highly controllable drug delivery by synergistic function of the targeting moieties could be accomplished when compared to conventional targeting strategies [28].

Therefore, with the aim of enhancing thrombolysis efficacy, we postulated that a dual targeted rtPA nano-drug would be feasible to achieve better thrombolytic outcomes for translation into a clinical setting. When compared with the single targeting strategy, nanoparticles engineered with dual targeting mechanism, either augment the single targeting ligand with a second ligand or with magnetic targeting, has successfully been used for cancer therapy, but it has never have been demonstrated for targeted thrombolysis in vivo. Because dual-targeting nanoparticles were shown to increase differentiation between cancer and normal cells and lead to enhanced accumulation of the drug in tumors [29], the same principle is deemed to be feasible for more selectively targeted delivery of thrombolytic agents to thrombus. Specifically, magnetic PLGA nanoparticles have been explored in a dual targeted delivery of paclitaxel and curcumin in brain tumor therapy [30] and protein antigen delivery for immune stimulation [31].

**Figure 1:**
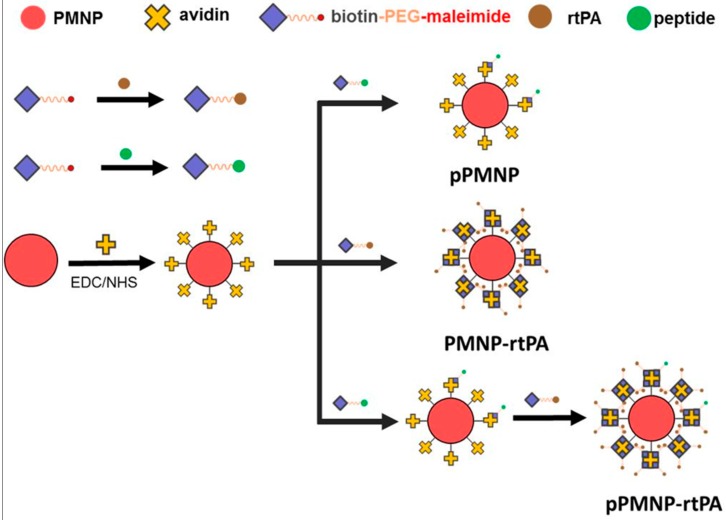
The schematic diagram showing the preparation of peptide conjugated poly(lactic-co-glycolic acid) (PLGA) magnetic nanoparticles (pPMNP), recombinant tissue plasminogen activator (rtPA) conjugated PLGA magnetic nanoparticles (PMNP-rtPA), and peptide/rtPA conjugated PLGA magnetic nanoparticles (pPMNP-rtPA).

In the present study, PLGA MNP (PMNP) was prepared to encapsulate Fe_3_O_4_ nanoparticles in a single oil-in-water emulsion step. Thrombolytic agent (rtPA) and fibrin-affinity peptide (GPRPPGGSKGC) were conjugated to PMNP surface that was modified with avidin to prepare pPMNP-rtPA, through avidin-biotin interaction and thiol–maleimide “click” reactions using biotin-PEG-maleimide (Figure 1). The physico-chemical properties of nanoparticles during preparation were analyzed, followed by biocompatibility and thrombolysis analysis in vitro. Finally, the clot thrombolysis efficiency of pPMNP-rtPA was tested in vivo in a rat embolic model.

## 2. Results and Discussion

### 2.1. Preparation of Peptide and rtPA Conjugated PLGA Magnetic Nanoparticles

Iron oxide MNP are prone to agglomeration in biological fluids due to high surface energy and the tendency to adsorb proteins, which might limit their in vivo application. After entrapping in a polymer matrix, the MNP improve stability in biological fluids and increase the half-life during circulation in vivo [32]. Therefore, in this study, PLGA was used as a polymer matrix to encapsulate hydrophobic oleic acid-coated iron oxide MNP (OMNP) and form PLGA magnetic nanoparticles (PMNP) in a single oil-in-water (O/W) emulsion step in order to prevent aggregation of iron oxide MNP. After modifying PLGA surface with avidin through carbodiimide-mediated covalent bond formation between the carboxyl groups of PLGA and the amine groups of avidin, avidin-biotin interaction was explored for facile synthesis of rtPA and/or peptide conjugated nanoparticles (Figure 1). Avidin is a protein showing considerable affinity for biotin, which is an important co-factor in many biological processes. Avidin has the ability to bind up to four biotin molecules with the strongest non-covalent interaction being known to exist between a protein and its ligand (K_d_ = 10^−15^ M) [33]. In addition, the bond formation between biotin and avidin is very rapid and the formed bond is unaffected by pH, organic solvents, or temperature, which facilitates the conjugation of rtPA (or peptide) to PMNP-avidin. The one-to-four binding ratio of avidin toward biotin also allows high loading of rtPA on nanoparticle surface in a controllable manner to exert high thrombolytic activity.

Therefore, we used biotin as a mediator in this study to introduce rtPA and peptide to PMNP-avidin. This was accomplished using biotin-PEG-maleimide, which could interact with the nanoparticle through avidin/biotin interaction in one end and conjugate rtPA (or peptide) at the other using thiol–maleimide “click” chemistry [34]. It is well known that maleimide specifically reacts with the sulfhydryl group between pH 6.5 to 7.5 and forms a stable irreversible thioether linkage. It should be noted, however, a more alkaline conditions (pH > 8.5) favors reaction with primary amines and the rate of maleimide hydrolysis increases [35]. The facile conjugation rtPA and peptide to PMNP-avidin is through binding of rtPA (or peptide) to biotin-PEG-maleimide with a 1:1 molar ratio of maleimide: rtPA (or peptide). This could be accomplished, as either GPRPPGGSKGC or rtPA has only one thio (–SH) group from the cysteine (C) residue at one end of the peptide or an unpaired cysteine residue in rtPA (contains 35 cysteine residues and 17 disulfide linkages) [36]. 

**Figure 2:**
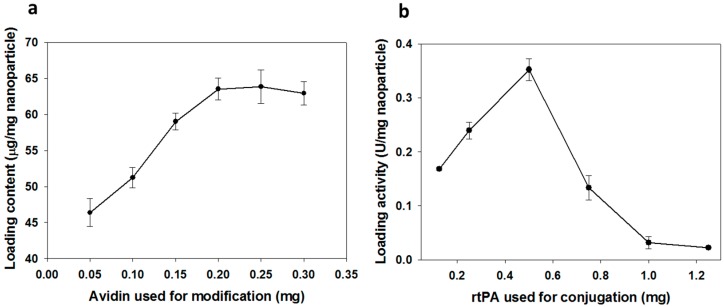
The effect of avidin and rtPA used during for immobilization on the loading of avidin protein (**a**) and rtPA activity (**b**).

It is imperative to first modify PMNP surface with vast amount of avidin for conjugating with abundant biotin-PEG-rtPA in order to optimize the preparation of pPMNP-rtPA. This high specific fibrinolytic activity of immobilized rtPA per unit weight of nanoparticle will render the use of minimum amount of nanoparticles for effective thrombolysis in vivo. For this purpose, the influence of the amount of avidin used for immobilization on the amount of immobilized avidin protein per mg of nanoparticle was studied. The immobilized avidin content increased with the amount of avidin used during the modification step and reached a plateau at around 0.2 mg avidin, as shown in Figure 2a. Therefore, the optimum avidin modification condition is reached when 0.2 mg avidin is reacted with 1 mg PMNP for preparation of PMNP-avidin where 63.5 ± 1.5 μg avidin/mg PMNP loading content could be achieved. Using this PMNP-avidin preparation, we proceeded to study effect of the amount of rtPA used for immobilization on the amidolytic activity of immobilized rtPA activity per mg of nanoparticle. The loading activity increases with rtPA used for conjugation until reaching 0.5 mg rtPA and decreases thereafter, as shown in Figure 2b. Therefore, the optimum drug loading is reached when 0.5 mg rtPA is reacted with 1 mg PMNP-avidin to fabricate PMNP-rtPA with 0.35 ± 0.02 U rtPA/mg PMNP-avidin loading activity. For the proper preparation of pPMNP-rtPA without overloading PMNP-avidin, PMNP-avidin was first reacted with biotin-PEG-peptide corresponding to 5 mol% of rtPA in the first step, followed by reacting with biotin-PEG-rtPA to fabricate pPMNP-rtPA (Figure 1). The final loading of peptide on pPMNP-rtPA was 0.46 ± 0.03 mg/mg nanoparticle, while the rtPA activity slightly reduced to 0.32 ± 0.01 U rtPA/mg nanoparticle.

**Figure 3:**
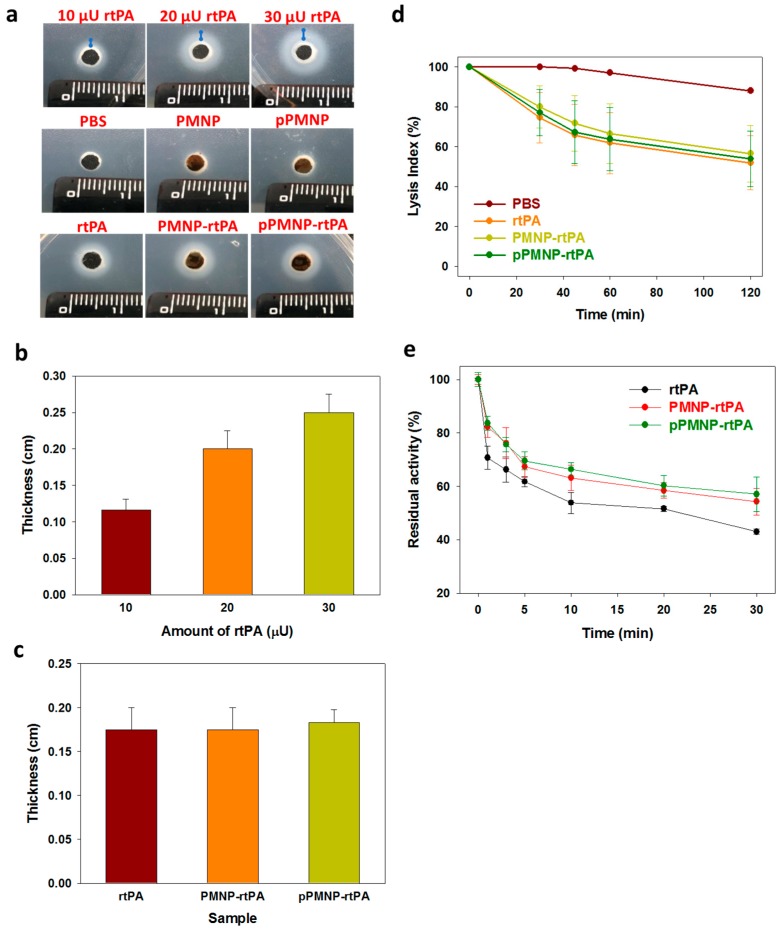
The clot lysis ability of free and immobilized rtPA form the fibrinolytic activity assay using the agar plate. Fibrinolysis activity was observed from a clear zone around the sample hole after incubation at 37 °C for 24 h using free rtPA at 10, 20, and 30 μU and PMNP-rtPA and pPMNP-rtPA at 20 μU (**a**). The thickness of the clear zone (blue lines) in (**a**) was measured and compared between free rtPA at different dosage (**b**), and between free rtPA, PMNP-rtPA, and pPMNP-rtPA (**c**). (**d**) Comparison of the fibrinolytic activity of phosphate buffered saline (PBS) (control), rtPA, PMNP-rtPA and pPMNP-rtPA from the lysis index using thromboelastometry at 1.7 μU/mL rtPA dosage. (**e**) The inhibition of free rtPA, PMNP-rtPA, and pPMNP-rtPA by plasminogen activator inhibitor-1 (PAI-1) in vitro by measuring the residual amidolytic activity of rtPA after incubating with PAI-I at 37 °C for different times.

The activity of rtPA determined above is the amidolytic activity (U) to hydrolyze a low molecular weight substrate (S-2288), but not associated with the clot lysis thrombolytic activity. The fibrinolytic property of free and immobilized rtPA was compared from the agar plate assay to show the thrombin-mediated fibrinolytic activity of rtPA for application in thrombolysis in order to ascertain the retention of biological activity of rtPA, which is likely to change and diminish the clot lysis activity due to protein denaturation [37]. To validate this assay method, the dosage dependent sensitivity was first confirmed by determining the thickness of clear zones around sample holes, containing different amount of rtPA, due to the enzymatic action of the drug. The thickness of a clear zone around the sample hole of the solidified agar, due to lysis of fibrin gel with rtPA enzymatic function, increased with the amount of rtPA used for the assay, as shown in Figure 3a,b. When PMNP-rtPA or pPMNP-rtPA was placed in the sample hole on the agar plate, the size of the clear zone created by the action of immobilized rtPA was equal to that created by free rtPA at the same drug dosage (Figure 3a,c), endorsing that rtPA conjugated to PMNP and pPMNP could fully retain its lytic action against fibrin clot. Most importantly, nano-carrier conjugated with the peptide ligand (pPMNP-rtPA) showed the same clot lysis activity when compared to the non-conjugated one (PMNP-rtPA), implying that modifying PMNP with the targeting peptide ligand would not influence the thrombolysis activity of rtPA. The absence of any clear zone around the phosphate buffered saline (PBS), PMNP and pPMNP-containing sample hole shows that they have no fibrinolytic activity to hydrolyze the fibrin and the lysis of agar plate is solely due to the fibrinolytic activity of rtPA (Figure 3a).

We further used thromboelastometry to determine blood clots lysis induced by rtPA by measuring the lysis index, which was shown to be dosage-dependent on free rtPA concentration used for the assay, to evaluate the thrombolytic effects [38]. As shown in Figure 3d, there is no significant difference in lysis index among free rtPA, PMN-rtPA and pPMNP rtPA irrespective of assay time (30, 45, 60 and 120 min) at the same rtPA drug dosage, which is in accordance with those from agar plate assays (Figure 3c). Taken together, both assays confirm the full retention of rtPA fibrinolytic activity after conjugation to PMNP, irrespective of peptide conjugation by using the pre-designed conjugation step as shown in Figure 1, which facilitate the use of pPMNP-rtPA for dual targeted rtPA delivery. 

The plasminogen activator inhibitor-1 (PAI-1) is the main inhibitor of circulating rtPA and inactivation of rtPA by PAI-1 is the main cause for the short half-life of rtPA [39]. Therefore, it will be highly desirable that the PMNP-rtPA and pPMNP-rtPA developed in this study will not only retain its fibrinolytic activity (Figure 3c), but also prolong the circulation time of rtPA. Therefore, we used the chromogenic substrate S-2288 to determine the amidolytic activity of PMNP-rtPA and pPMNP-rtPA when pre-incubated with PAI-1 for different times and compared with that of free rtPA. The immobilized rtPA was inactivated by PAI-1 more slowly than free rtPA, indicating that the rtPA in PMNP-rtPA and pPMNP-rtPA was protected from PAI-1 to some extent, as shown in Figure 3e. This might be due to the effects of steric hindrance of the nano-carrier for accessibility of PAI-1 to bind rtPA or due to the inaccessibility of the PAI-1-binding site in the bound rtPA [40].

### 2.2. Characterization of Physico-Chemical Properties

**Figure 4:**
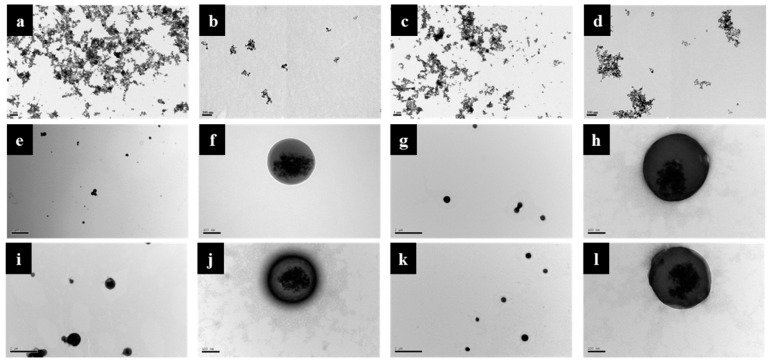
The transmission electron microscope (TEM) images of Fe_3_O_4_ magnetic nanoparticles (MNP) (**a**,**b**), oleic acid coated magnetic nanoparticles (OMNP) (**c**,**d**), PLGA magnetic nanoparticles (PMNP) (**e**,**f**), avidin-modified PLGA magnetic nanoparticles (PMNP-avidin) (**g**,**h**), rtPA-conjugated PLGA magnetic nanoparticles (PMNP-rtPA) (**i**,**j**), and peptide/rtPA-conjugated PLGA magnetic nanoparticles (pPMNP-rtPA) (**k**,**l**). Bar = 2 μm (**e**,**g**,**i**,**k**), 1 μm (**a**,**c**) and 100 nm (**b**,**d**,**f**,**h**,**j**,**l**).

From the transmission electron microscope (TEM) images that are shown in Figure 4a–d, iron oxide MNP and OMNP agglomerated in water at low magnification due to Van der Waals force or weak magnetic attractions from magnetic dipole–dipole interactions [41]. Nonetheless, the average particle size of discrete nanoparticles could still be estimated to be around 10 nm from high magnification micrographs and consistent with similar researches that also used co-precipitation method to prepare iron oxide MNP [42]. After the encapsulation of OMNP in PLGA, PMNP shows smooth and spherical surface morphology of ~300 nm diameter and an agglomerate core of iron oxide MNPs (Figure 4e,f). This indicates that all OMNP were well encapsulated within the PLGA matrix after a single oil-in-water (o/w) emulsion/solvent evaporation step. Modification with avidin (PMNP-avidin), conjugation with rtPA (PMNP-rtPA), and conjugation with peptide (pPMNP-rtPA) do not lead to much change of particle size and surface morphology (Figure 4g–l). Nonetheless, a gray layer around the round contours from 1% phosphotungstic acid staining, which binds to the basic groups (lysine and arginine residues) of proteins, suggests that both avidin and rtPA were successfully immobilized on the nanoparticle surface. 

**Figure 5:**
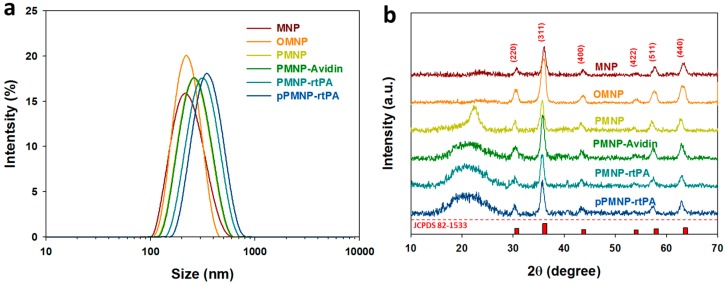
Characterization of Fe_3_O_4_ magnetic nanoparticles (MNP), oleic acid coated magnetic nanoparticles (OMNP), PLGA magnetic nanoparticles (PMNP), avidin-conjugated PLGA magnetic nanoparticles (PMNP-avidin), rtPA-conjugated PLGA magnetic nanoparticles (PMNP-rtPA), and peptide/rtPA-conjugated PLGA magnetic nanoparticles (pPMNP-rtPA) by dynamic light scattering (DLS) (**a**) and X-ray diffraction (XRD) (**b**).

From the dynamic light scattering (DLS) results that are shown in Figure 5a, the average hydrodynamic diameters for MNP, OMNP, PMNP, PMNP-avidin, PMNP-rtPA, and pPMNP-rtPA are 230.7, 229.4, 252.7, 278.8, 291.2, and 321.1 nm, respectively (Table 1) and consistent with the trend observed from TEM (Figure 4). The particle size constantly increased during the sequential modification/conjugation steps from PMNP to pPMNP-rtPA due to increasing amount of molecules coated on nanoparticle surface. The polydispersity index (PDI) were all below 0.22 (Table 1), indicating that the particle size distribution is uniform for all samples, and every sample has good suspension stability [43]. 

From electrophoretic mobility measurements, the average zeta potential of MNP is positive at 12.1 mV, due to residual ammonium ions associated with the particles as ammonia was used for the co-precipitation of ferric and ferrous ions during the synthesis (Table 1). This value changed to negative values for OMNP (−17.6 mV) and PMNP (−30 mV) due to the carboxylic group of oleic acid and PLGA. The zeta potential shifted to −25.8 mV after avidin modification as the isoelectric point (pI) of this protein is approximately 10 [44]. A similar effect due to the positive change of rtPA (pI = 7.6) and peptide (pI =10) slightly changed the zeta potential to −24.4 mV (PMNP-rtPA) and −22.1 mV (pPMNP-rtPA). 

The x-ray diffraction (XRD) patterns that are shown in Figure 5b reveal six characteristic peaks at 2θ = 30.3°, 35.7°, 43.4°, 53.87°, 57.4°, and 63.1° for all samples, which represent the (220), (311), (400), (422), (511), and (440) planes of a cubic cells and corresponds to the magnetite structure (JCPDS file no. 82-1533). This confirms that all of the nanoparticles were pure Fe_3_O_4_ with a spinel structure and PLGA encapsulation or surface conjugation steps did not lead to phase change of Fe_3_O_4_. The average crystal size calculated from the Scherrer equation, using the highest diffraction peak at 2θ = 35.7°, with the XRD line broadening and assuming spherical crystals, ranged from 10 to 12 nm (Table 1), supporting the results from TEM. 

**Figure 6:**
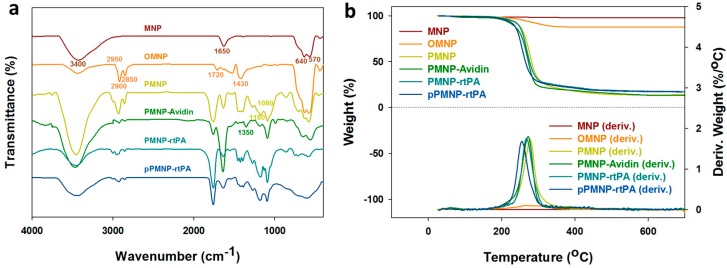
Characterization of Fe_3_O_4_ magnetic nanoparticles (MNP), oleic acid coated magnetic nanoparticles (OMNP), PLGA coated magnetic nanoparticles (PMNP), avidin-conjugated PLGA coated magnetic nanoparticles (PMNP-Avidin), rtPA-conjugated PLGA coated magnetic nanoparticles (PMNP-rtPA) and peptide/rtPA-conjugated PLGA magnetic nanoparticles (pPMNP-rtPA) by Fourier transform infrared (FTIR) spectroscopy (**a**) and thermogravimetric analysis (TGA) (**b**).

The Fourier transform infrared (FTIR) spectra are shown in Figure 6a for all samples. The characteristic peak of Fe_3_O_4_ MNP at 640 and 570 cm^−1^ represented the Fe–O bond while the peak in the 3200 to 3600 cm^−1^ range and at 1650 cm^−1^ might represent the N–H bond contributed from residual ammonia, as observed from its positive zeta potential (Table 1). For OMNP, the characteristic peak at 2950, 2900, and 2850 cm^−1^ represented the C–H bond of –CH, –CH_2_, and –CH_3_, respectively. The characteristic peak that ranged 1750–1680 cm^−1^ represented the C=O (carboxylic acid) bond, while the characteristic peak ranged 1450–1400 cm^−1^ represented the O–H (carboxylic acid) bond and the C–H bond, supporting oleic acid coating. For PMNP, the characteristic peak at 1180 cm^−1^ and 1080 cm^−1^ represented the C–O bond of ester and alcohol, indicating the successful entrapment of OMNP in PLGA. A new characteristic peak at 1350 cm^−1^ that was assigned to the C–N (acryl) bond was observed for PMNP-avidin, supporting surface modification of PMNP with avidin. No new peaks could be identified after peptide and rtPA conjugation, as expected. 

The thermogravimetric analysis (TGA) results that are shown in Figure 6b reveal weight loss starting from 270 °C for OMNP compared with MNP due to the decomposition of the organic oleic acid molecules on Fe_3_O_4_ MNP and the final residual weight at 700 °C indicates 10% (*w/w*) oleic acid in OMNP (Table 2). For PLGA-encapsulated nanoparticles, substantial weight loss near 275 °C was evident due to PLGA decomposition and the residual weights of PMNP, PMNP-avidin, PMNP-rtPA, and pPMNP-rtPA at 700 °C were approximately 14.0%, 13.4%, 17.1%, and 16.9%, respectively (Table 2). For comparison, the Fe_3_O_4_ content that was determined from inductively coupled plasma-optical emission spectrometry (ICP-OES) for MNP, OMNP, PMNP, PMNP-avidin, PMNP-rtPA, and pPMNP-rtPA are 97.8%, 87.1%, 13.5%, 12.3%, 10.5%, and 10.1%, respectively (Table 2). Separate experiment indicates the residual weight of PLGA, avidin, and rtPA after burning to 700 °C in nitrogen were 0%, 20.1%, and 33.7%, respectively, due to their respective synthetic and natural material characteristics (data not shown). When considering the residual weight of avidin and rtPA, the Fe_3_O_4_ content from TGA and ICP-OES experiments could be deemed to be consistent. 

**Figure 7:**
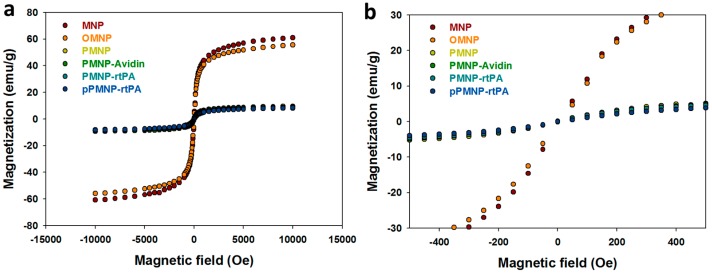
Characterization of Fe_3_O_4_ magnetic nanoparticles (MNP), oleic acid coated magnetic nanoparticles (OMNP), PLGA coated magnetic nanoparticles (PMNP), avidin-conjugated PLGA coated magnetic nanoparticles (PMNP-avidin), rtPA-conjugated PLGA coated magnetic nanoparticles (PMNP-rtPA), and peptide/rtPA-conjugated PLGA magnetic nanoparticles (pPMNP-rtPA) by superconducting quantum interference device (SQUID) hysteretic magnetization curves (**a**). The remnant (residue) magnetization of all samples is close to zero, as shown in (**b**).

Figure 7a shows the magnetization curves of all samples from superconducting quantum interference device (SQUID) analysis at room temperature. The remnant (residue) magnetization (without magnetic field) was determined from Figure 7b to determine the superparamagnetic property. The remnant magnetization of MNP is close to zero (0.21 emu/g), endorsing its superparamagnetic property and consistent with its ~10 nm size from TEM and XRD experiments and in accordance with reported extremely low or non-existent remanence of Fe_3_O_4_ NMP [45]. The remnant magnetization values of OMNP, PMNP, PMNP-avidin, PMNP-rtPA, and pPMNP-rtPA are also close to zero (< 0.3 emu/g) (Table 2), indicating MNP retained the characteristic superparamagnetism behavior during the synthesis of PLGA-based nanoparticles. The superparamagnetism behavior refers that all magnetic nano-carriers can be magnetized when an external magnetic field is applied and there is no residual magnetic interaction after removing the magnetic field. This behavior depends on the size of iron oxide MNP and it generally occurs below ~20 nm particle size, which supports the characterizing results from TEM and XRD. Undoubtedly, the use of superparamagnetic pPMNP-rtPA confers an important property for in vivo application as the drug-conjugated magnetic nano-carrier could be easily dispersed after guidance to the target site by removing the applied magnetic field, which can prevent undesirable particle agglomeration and possible vessel blockage [46]. The saturation magnetization value of OMNP reduced to 90% of that of MNP, consistent with the trend of MNP content from TGA or ICP-OES. This value reduced drastically to ~15% of that of MNP for all PLGA-encapsulated nanoparticles (i.e., PMNP, PMNP-avidin, PMNP-rtPA, and pPMNP-rtPA), which is due to the reduced MNP weight in the nanoparticles. Although the magnetization value is deemed to be sufficient for magnetic guidance in magnetically targeted thrombolytic drug delivery [47], dual targeting through synergistic ligand targeting will undoubtedly improve the drug targeting and clot lysis efficacy. 

### 2.3. In Vitro Biocompatibility

**Figure 8:**
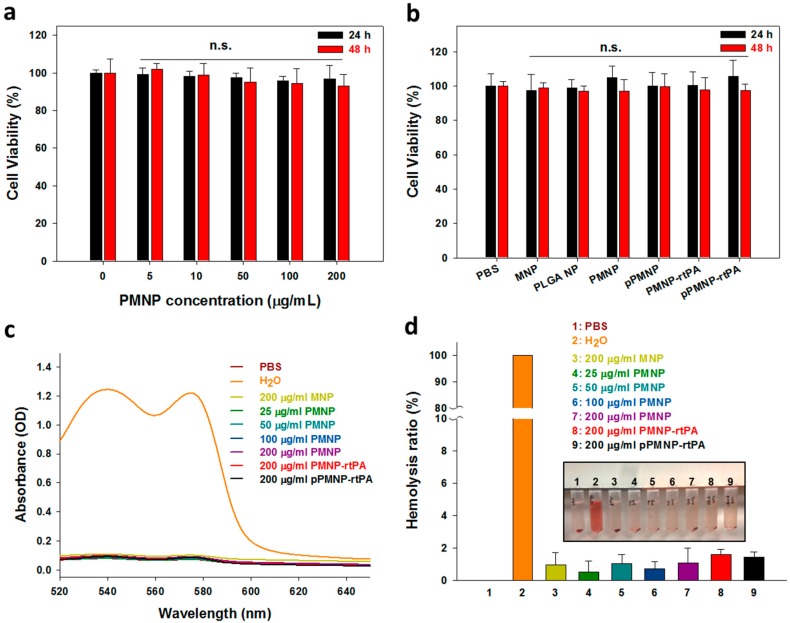
Cytocompatibility of PMNP at different concentrations (n.s.: not significant compared to 0 μg/mL at 24 or 48 h, all samples) (**a**) and of different nanoparticles at 200 μg/mL (n.s.: not significant compared to PBS at 24 or 48 h, all samples) (**b**) by MTT assay after contacting with NIH 3T3 cells. Hemocompatibility of different nanoparticles was determined from the hemolysis assay by incubation with diluted red blood cells in PBS at 37 °C for 2 h to obtain the full-wavelength absorption spectra of the supernatant (**c**) and the hemolysis ratio from OD_540_ (**d**). Water and PBS were used as the positive and the negative controls, respectively.

The biocompatibility was first determined from the cytocompatibility of PMNP at different concentrations after being incubated with NIH 3T3 fibroblasts for 24 and 48 h. The relative cell viability after contacting with PMNP shows no significance difference from that of control (without PMNP) up to 0.2 mg/mL, indicating PMNP is not toxic, even at concentrations suitable for in vivo injection, as shown from Figure 8a. Using this concentration, we further tested the cytotoxicity of all nanoparticles during pPMNP-rtPA synthesis. The relative cell viability of all samples not only remained above 70% at both time points, to conform with the cytocompatibility standards of ISO 10993-5, no significance difference was found for all groups when compared to the control (PBS), as shown in Figure 8b. 

Other than cytocompatibility, further biocompatibility consideration is through hemocompatibility to determine whether nanoparticles may induce hemolysis when administrated through intravenous injection. After incubation with purified rat red blood cells diluted in PBS at 37 °C for 2 h, the absorption spectra of the supernatant of MNP, pPMNP, PMNP-rtPA, and pPMNP-rtPA was the same as PBS (negative control), but not water (positive control), due to busting of red blood cells and release of oxyhemoglobin to show absorbance maxima at 540 and 577 nm (Figure 8c). The hemolysis effect was further examined through a quantitative comparison of the hemolysis ratio by using the difference in OD_540_ between the sample and the negative control (PBS) divided by the difference between the positive control (water) and the negative control. No visible hemolysis was observed between all samples and PBS, but not water, and the hemolysis ratio was lower than 2% for every nanoparticle (Figure 8d), indicating the good hemocompatibility of the nanoparticles synthesized according to the hemocompatibility standards of ASTM F756–08. Taken together, the good cytocompatibility and negligible hemolytic activity of pPMNP-rtPA underlines its excellent biocompatibility for in vivo application [48].

### 2.4. Targeting Effects

**Figure 9:**
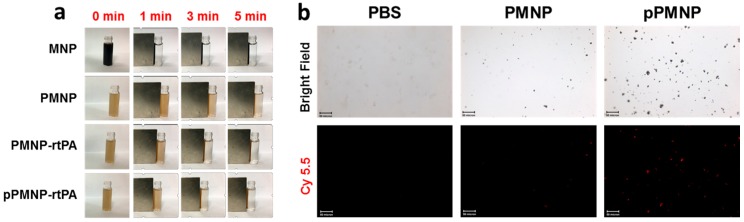
(**a**) Magnetic field guiding efficiency of different magnetic nanoparticles by attaching a magnet (1700 gauss) to the side of a tube containing 1 mg/mL nanoparticle in PBS for 5 min. (**b**) Fibrin-binding efficiency of PLGA magnetic nanoparticles (PMNP) and peptide-conjugated PLGA magnetic nanoparticles (pPMNP) was determined by contacting Cy 5.5-labelled magnetic nanoparticles (0.1 mg/mL) with a fibrin plate for 10 min. The fluorescence signal from the remnant nanoparticles bound to fibrin was observed under an inverted fluorescence microscope after washing (bar = 50 um).

The magnetic targeting effect was examined by magnetic guiding experiments by visually observing the solution turbidity after placing a magnet at the side of a tube containing 1 mg/mL nanoparticle for 5 min From Figure 9a, the time-lapsed change of supernatant turbidity indicated a magnetic field that was created by using a 1700 gauss magnet was effective to guide magnetic nanoparticles synthesized in this study to tube side against the gravity force, which reveals the possibility of using pPMNP-rtPA for magnetically guided thrombolysis. We used pre-synthesized Cy 5.5-encapsulated magnetic nanoparticles to demonstrate the fibrin binding ability of pPMNP and the ligand targeting effect. The fluorescently labelled nanoparticles were placed in a fibrin plate, shaken for 10 min, washed, and examined under an inverted fluorescence microscope to reveal the difference in binding ability between PMNP and pPMNP toward fibrin. In contrast to control with PBS showing no background fluorescence, Cy 5.5-labelled PMNP showed minimum fluorescence signal due to non-specific binding, in contrast to abundant scattered fluorescence associated with pPMNP due to specific peptide-fibrin biding, as shown in Figure 9b. The peptide GPRPPGGSKGC used here has been conjugated to fluorescently labeled MNP and showed high affinities for thrombi for multimodal fluorescence and magnetic resonance imaging of intravascular thrombus [49]. Therefore, the peptide-conjugated magnetic nanoparticles (pPMNP) offer the possibility for ligand-mediated fibrin-targeted thrombolytic drug delivery to induce thrombolysis. When compared with using monoclonal antibody against fibrin as a targeting ligand, the peptides are smaller in size, easier to produce than monoclonal antibodies, less likely to induce any immunologic reaction, but still in most cases, show high specificity and binding constants as monoclonal antibodies [50].

### 2.5. In Vitro Thrombolysis

**Figure 10:**
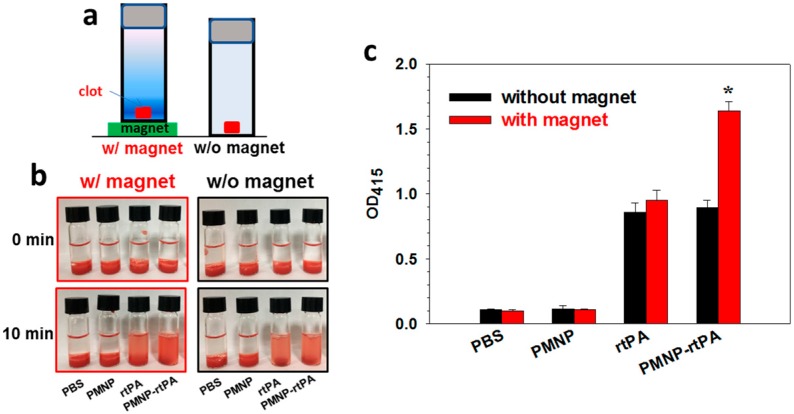
The in vitro static clot lysis with or without magnetic guidance in vertical position was carried out as illustrated in (**a**). The solution appearance after blood clot lysis (**b**) and the solution absorbance measured at 415 nm (OD_415_) (**c**) after incubating the blood clot with PBS, PMNP, rtPA or PMNP-rtPA solution (25 μU rtPA). * *p* < 0.05 compared with pPMNP-rtPA without magnet.

Static in vitro thrombolysis in vertical position, using a blood clot made from rat whole blood, was performed with or without a magnet, to determine the effectiveness of magnetic targeting in blood clot lysis (Figure 10a) [51]. For this purpose, the extent of clot lysis when pre-formed blood clot was treated by rtPA and PMNP-rtPA in a sample tube was compared under the influence of downward magnetic pulling force generated from a magnet placed at the bottom of the tube. The clots treated in PBS and PMNP were taken as controls. Blood clot lysis was monitored spectrophotometrically by measuring the absorbance of the supernatant at 415 nm (OD_415_) after 10 min. In PBS or PMNP without rtPA, the blood clot remains intact with the solution transparency similar to 0 min, as observed from Figure 10b. Nonetheless, the contact with rtPA or PMNP-rtPA resulted in red-colored supernatant solution due to release of red blood cell residues from the tight fibrin network in the blood clot. By comparing the OD_415_ values presented in Figure 10c, there is no significant difference in OD_415_ for rtPA and PMNP-rtPA without magnet, as observed before from the agar plate clot lysis assays (Figure 3c). Most importantly, only PMNP-rtPA, but not rtPA, shows magnet-sensitive lysis activity. The OD_415_ increased significantly (1.83 folds) due to a downward magnetic force being generated by the magnet to attract PMNP-rtPA toward the blood clot for enhanced fibrinolysis. 

**Figure 11:**
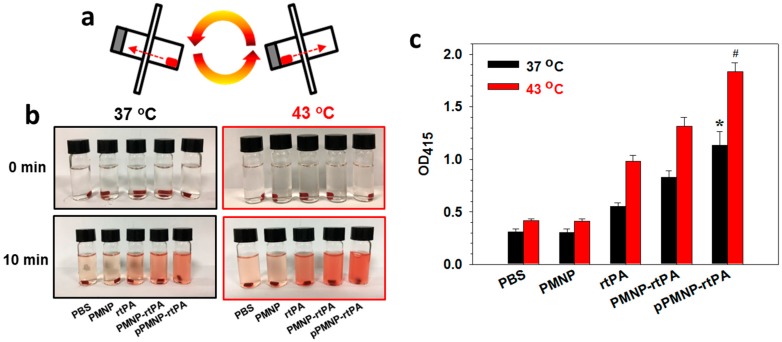
The in vitro dynamic clot lysis was carried out as illustrated in (**a**). The solution appearance after blood clot lysis (**b**) and the solution absorbance measured at 415 nm (OD_415_) (**c**) after incubating the blood clot with PBS, PMNP, rtPA, PMNP-rtPA, or pPMNP-rtPA solution (50 μU rtPA activity). * *p* < 0.05 compared with PMNP-rtPA at 37 °C, ^#^ *p* < 0.05 compared with PMNP-rtPA at 43 °C.

Dynamic in vitro thrombolysis was employed to verify enhanced clot lysis due to the conjugated peptide ligand at 37 °C and 43 °C by slowly rotating the clot-containing vial (Figure 11a). The mixing of a blood clot with the nanocarrier was deemed to be necessary for showing the ligand targeting effect of pPMNP-rtPA originated from fibrin binding. As in static test, the color intensity of the red-colored supernatant solution increased only for rtPA, PMNP-rtPA, and pPMNP-rtPA with the thrombolytic action of rtPA releasing red blood cell residues from the clot (Figure 11b). Most importantly, a significant difference in OD_415_ was found between pPMNP-rtPA and pPMNP-rtPA, regardless of temperature (Figure 11c). This underscores the importance of using fibrin-avid peptide moiety to upregulate the clot lysis activity of immobilized rtPA, due to the preferential binding to fibrin (Figure 9b). That temperature-dependent lysis was only being shown in rtPA-containing samples further supports rtPA-induced clot lysis, with higher rtPA enzymatic activity at 43 °C. 

**Figure 12:**
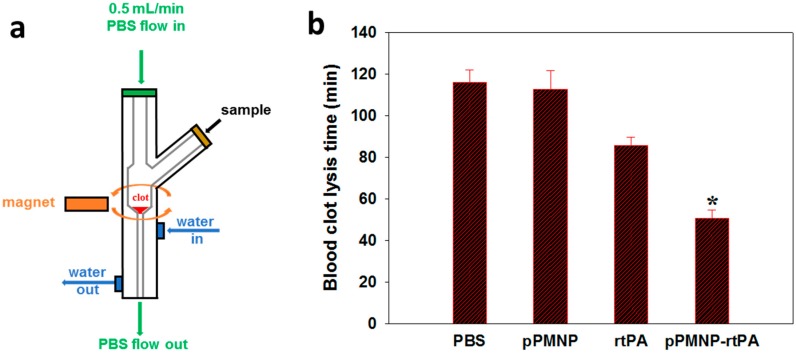
The schematic diagram of a flow model to evaluate pressure-driven thrombolysis at 0.5 mL/min (**a**). At time zero, different sample (PBS, pPMNP, rtPA, or pPMNP-rtPA) was introduced into the flow system and the blood clot lysis time was recorded when reperfusion occurs to determine the lysis efficiency (**b**). * *p* < 0.05 compared with rtPA.

After thrombolysis testing in a closed system, we studied simulated vascular embolization induced by a blood clot and pressure-driven clot lysis in a flow system (Figure 12a). The best pPMNP-rtPA sample was tested in the flow thrombolysis model at 37 °C while using a water jacketed glass tube. A blood clot was placed at the bottom of a tube with reduced diameter and lodged tightly within the tube inner circumference, in order to restrict lysis from the clot surface, and the sample was injected from a side opening. A magnet was placed below the clot, outside of the tube to introduce magnetic guidance. A fluid pressure gradient was generated with PBS flow from the top at 0.5 mL/min using a syringe pump. At time zero, a different sample was introduced into the flow system and the reperfusion time for clot dissolution (clot lysis time) was compared among different treatment groups to determine the lysis efficiency. The blood clot lysis efficiency of pPMNP-rtPA treatment was enhanced with significantly reduced clot lysis time (51 min), when reperfusion and disengaging of clot from the tube occurs. This could be compared with free rtPA (86 min) as shown in Figure 12b. The combination of magnetic and ligand targeting resulted in the shortest reperfusion time with 2.3-fold increase in lysis efficiency when compared to the pPMNP group.

### 2.6. In Vivo Thrombolysis

**Figure 13:**
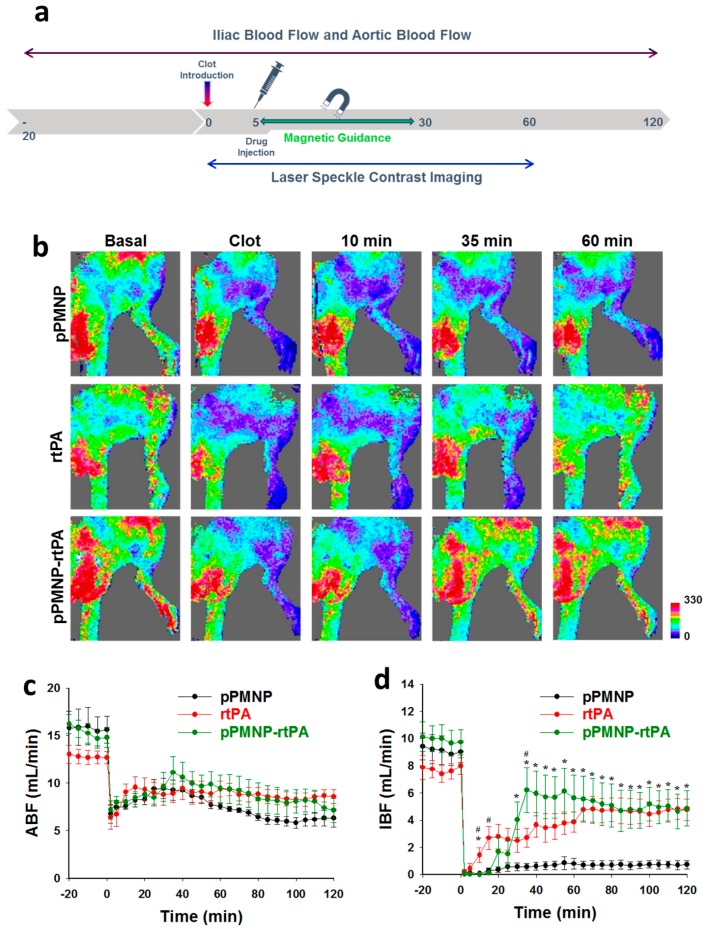
The in vivo thrombolytic effects of pPMNP-rtPA by evaluating targeted thrombolysis in a rat embolic model. (**a**) A blood clot was lodged into the left iliac artery and after 5 min pPMNP (*n* = 5), rtPA (1.5 U/kg; *n* = 5) or pPMNP-rtPA (0.3 U/kg; *n* = 5) was delivered from the right iliac artery and subject to magnetic guidance for 25 min. (**b**) Representative images of blood perfusion rate in the hind limb area at different time points after clot introduction using a laser speckle contrast imager. The abdominal aortic blood flow (ABF) (**c**) and iliac blood flow (IBF) (**d**) before and after blood clot introduction (0 min) was determined from ultrasonic flow probes. * *p* < 0.05 rtPA or pPMNP-rtPA vs. PMNP, ^#^ *p* < 0.05 rtPA vs. pPMNP-rtPA.

The clinical application potential of dual targeted delivery of rtPA was evaluated by determining the in vivo fibrinolytic efficacy of pPMNP-rtPA where induce targeted thrombolysis under magnetic guidance was assessed in a rat embolic model [48]. Five minutes after lodging the left iliac artery upstream of the pubic epigastric artery with a blood clot, pPMNP (vehicle), free rtPA, or pPMNP-rtPA was administered intra-arterially via the right iliac artery. Magnetic guidance with a 0.5 T magnet started immediately after drug administration. This was repeated in five successive cycles (5 min each) from the branch of abdominal aorta and iliac artery to the branch of iliac artery and the femoral artery (1 min) and to the lower extremities (4 min) (Figure 13a). The blood flow pattern changes around the surrounding areas of the hind limb was acquired using a laser speckle contrast imager, while the rates of iliac blood flow (IBF) and aortic blood flow (ABF) were determined using ultrasonic flow probes.

The hind limp perfusion reduced dramatically from the basal after introducing the clot, which created vascular thrombosis for evaluation of the fibrinolytic activity of rtPA in vivo, as shown in Figure 13b. It is evident that pPMNP-rtPA injection improved the blood perfusion in the hind limb 35 min post-treatment due to restoring the blood flow downstream of the blood clot site, which was not achievable in both pPMNP and rtPA groups. The rtPA group showed increased blood perfusion only after 60 min treatment to be similar to the pPMNP-rtPA, while the pPMNP group showed a similar thrombosis condition without any improvement of the perfusion rate (Figure 13b). The time-lapsed hemodynamics results, including ABF (Figure 13c) and IBF (Figure 13d), were shown after different treatments. Before clot lodging, the IBF values were 9.4 ± 1.0, 7.9 ± 0.9 and 10.1 ± 1.1 mL/min for pPMNP, rtPA, and pPMNP-rtPA, respectively. Nonetheless, the IBF values reduced to nearly 0% for all groups after thromboembolism, due to the complete occlusion of the left iliac artery. Simultaneously, the ABF also reduced to 6 mL/min in all groups. After the treatment, the IBF restored to 0.7 ± 0.3, 4.8 ± 0.8, and 4.9 ± 1.3 mL/min for pPMNP, rtPA, and pPMNP-rtPA, which was 8.1 ± 3.5%, 60.7 ± 8.8%, and 46.9 ± 10.8% of the respective initial value. From 10 to 35 min, there was a significant difference in IBF between rtPA and pPMNP-rtPA, as revealed from the perfusion image (Figure 13b). However, there was no significant difference in IBF between rtPA and pPMNP-rtPA from 40 min to 120 min, but both showed significant difference from pPMNP. In comparison, the ABF did not show dramatic recovery as IBF, although some recovery of ABF was also observed for the rtPA and pPMNP-rtPA groups after 100 min due to downstream restoration of IBF. Most importantly, as the dosage of pPMNP-rtPA was 0.3 U/kg compared to 1.5 U/kg for rtPA, we demonstrated comparable efficacy in restoring blood flow and in vivo thrombolysis using only 20% dosage of free rtPA. By allowing the use of a lower rtPA dosage with shortened clot lysis time, this dual targeted nanomedicine approach will be a feasible strategy for improving the efficacy and safety of rtPA in reducing bleeding risk [52]. Overall, the in vivo study suggested the clinical feasibility using dual targeted delivery strategy for rtPA and that dual targeted pPMNP is an efficient functional nano-carrier for comparable in vivo thrombolysis outcomes at reduced rtPA dosage, which might reduce the hemorrhagic side effects in clinical thrombolytic therapy.

## 3. Materials and Methods

### 3.1. Materials

1-(3-Dimethylaminopropyl)-3-ethylcarbodiimide hydrochloride (EDC), *N*-hydroxysuccinimide (NHS), iron (II) chloride tetrahydrate, and iron(III) chloride hexahydrate were purchased from Organics, Thermo Fisher Scientific (Geel, Belgium). Acetone, dichloromethane (DCM), oleic acid, polyvinyl alcohol (PVA) (hydrolyzed, M.W. 30,000–70,000), and plasminogen activator inhibitor-1 (PAI-1) were purchased from Sigma-Aldrich (St Louis, MO, USA). Poly(lactic-co-glycolic acid) (PLGA) (lactide/glycolide = 50/50, molecular weight = 15,000~30,000, intrinsic viscosity = 0.35 ± 0.05 dL/g) was purchased form Green Square Co. (Taoyuan, Taiwan). Recombinant tissue plasminogen activator (rtPA, Actilyse^®^) was provided form Boehringer Ingelheim (Ingelheim am Rhein, Germany). Avidin and biotin-PEG-maleimide (M.W. 3500) were purchased form Merck (Darmstadt, Germany) and JenKem Technology USA Inc. (Plano, TX, USA), respectively.

### 3.2. Preparation of Magnetic Nanoparticles

The oleic acid coated iron oxide magnetic nanoparticles (OMNP) were prepared by the co-precipitation method. FeCl_3_·6H_2_O (2.15 g) and FeCl_2_·4H_2_O (0.79 g) were dissolved in 50 mL ddH_2_O in a three necks glass reactor. Nitrogen was purged for 10 min to remove oxygen, followed by heating the solution to 60 °C. Five milliliter of NH_4_OH was added dropwise with a needle into the reactor and then reacted for 30 min under nitrogen by stirring at 500 rpm. The solution was cooled to room temperature while under continuous nitrogen purging. The magnetic nanoparticles was washed three times with distilled deionized water (ddH_2_O) after recovering with a magnet and the final solution pH was adjusted to 5 using 0.1 N HCl. The solution was sonicated for 10 min, followed by nitrogen purging for 10 min before heating to 60 °C. Ten milliliters of 2.77% (*v/v*) oleic acid solution prepared in acetone was then added dropwise with a needle into the reactor and reacted for 30 min under 500 rpm stirring. Nitrogen purge continued while cooling down the solution to room temperature. After removing acetone by evaporation, OMNPs was dispersed in chloroform to 1% (*w/w*) for storage. 

The OMNPs prepared above were entrapped in PLGA polymer matrix by the single emulsion-solvent evaporation method to prepare PLGA MNP (PMNP). Fifty milligram of PLGA was dissolved in 2.5 mL organic solvent mixture (2.25 mL acetone and 0.25 mL DCM), followed by sonicating for 10 min. Five hundred microliters of OMNPs prepared above (10 mg OMNPs in 1 mL chloroform) was added, vortexed for 1 min and sonicated for 1 min in a sonication bath. The mixture was quickly poured into 25 mL phosphate buffered saline (PBS), vortexed for 1 min and sonicated for 5 min (Q700, Qsonica, Newtown, CT, USA). The resulting solution containing PMNPs was added dropwise into 50 mL 0.3% (*w/v*) PVA solution prepared in PBS and stirred at 1000 rpm. After incubating at 30 °C for 12 h, the solution was placed in a rotary evaporator and then dried for 1 h at 30 °C for removing residual solvents and washed twice with ddH_2_O using magnetic decantation. To modify the surface of PMNPs with avidin, 1 mg/mL PMNPs in PBS (pH 7.4) was mixed with 0.5 mL EDC (0.5 mg/mL) and 0.5 mL NHS (0.5 mg/mL) prepared in 0.5 M pH 5 MES buffer and rotated at 6 rpm for 1 h at room temperature for the activation of the carboxyl groups in PLGA. After washing twice with PBS, activated PMNP was recovered by magnetic decantation and re-dispersed in 1 mL PBS, followed by adding 1 mL avidin (0.2 mg/mL) in PBS and mixed by rotation at 6 rpm for 3 h at room temperature. After washing twice with PBS, PMNP that was conjugated with avidin (PMNP-avidin) was recovered by magnetic decantation and re-dispersed in 1 mL PBS for immobilization of peptide/rtPA using biotin-PEG-peptide/biotin-PEG-rtPA through avidin-biotin interaction (Figure 1). To prepare biotin-PEG-peptide, 0.65 μg biotin-PEG-maleimide was mixed with 0.21 μg fibrin-avid peptide (GPRPPGGSKGC) in 1 mL PBS and then mixed at 4 °C for 24 h. Similarly, 27 μg biotin-PEG-maleimide was mixed with 0.5 mg rtPA in 1 mL PBS at 4 °C for 24 h to obtain biotin-PEG-rtPA. The solutions prepared above were mixed successively with PMNP-avidin, first by adding biotin-PEG-peptide to PMNP-avidin and mixed for 24 h at 4 °C, followed by adding biotin-PEG-rtPA and mixed for another 24 h at 4 °C. Finally, the particles were washed twice with PBS to obtain peptide/rtPA conjugated PLGA magnetic nanoparticles (pPMNP-rtPA). As a control, the peptide conjugated PLGA magnetic nanoparticles (pPMNP) or rtPA conjugated PLGA magnetic nanoparticles (PMNP-rtPA) were also prepared without adding biotin-PEG-peptide or biotin-PEG-rtPA during synthesis (Figure 1). 

### 3.3. Determination of rtPA and Peptide Conjugation

The amount of avidin loading on nanoparticles was measured by the Pierce™ BCA protein assay kit from Thermo Fisher Scientific (Geel, Belgium). All of the samples were collected after modification and dilute with PBS to appropriate concentration. Fifty microliter sample (1 mg/mL) and 1 mL BCA reagent were added into a 2 mL brown centrifuge tube and reacted for 30 min at 37 °C. After cooling to room temperature, the chromogenic product was measured by an UV-Vis spectrophotometer at 562 nm. The loading content of rtPA was determined as mg of protein per mg of nanoparticles. For rtPA loading, the activity of immobilized rtPA was spectrophotometrically determined from its amidolytic activity using S-2288 as a specific protease chromogenic substrate (Chromogenix, Mölndal, Sweden) following that manufacturer’s protocols. Two hundred microliter of samples (rtPA, PMNP-rtPA and pPMNP-rtPA) was diluted with 0.2 mL 0.1 M pH 7.4 Tris buffer and incubated at 37 °C for 5 min. Two hundred microliter of S-2288 substrate (1 mM) was added and vortexed immediately before incubating at 37 °C. To stop the enzymatic reaction, 0.1 mL acetic acid (20% (*v/v*)) was added after 30 s and solution absorbance was determined for the chromogenic product using an UV-Vis spectrophotometer at 405 nm (OD_405_) after magnetic separation. The amidolytic activity (U) was calculated as OD_405_ × 0.313 × 0.2/0.5 using respective nanoparticles as the blank.

### 3.4. Charactrization of Physico-Chemical Properties

The particle morphology of the prepared sample was analyzed using a transmission electron microscope (TEM, JEOL JEM2000 EXII, Tokyo, Japan) at an accelerating voltage of 100 kV. For OMNP, a diluted sample that was prepared in ethanol was used while other samples were prepared in ddH_2_O. After dropping a sample solution on the surface of cooper grids, it was dried in a 37 °C oven for 24 h and blotted with a filter paper. The sample was stained with 1% (*w/v*) phosphotungstic acid aqueous solution for 1 min before analysis. The particles size distribution was analyzed using a Zetasizer (Nano ZS 90, Malvern Instruments, Malvern UK) at 25 °C. The measurement was carried out by setting equilibration time at 30 s, measurement duration at automatic, number of measurement at 3, and delay between measurements at 5 s. The OMNP sample was prepared by dispersing in ethanol at 0.1 mg/mL, while other samples were prepared by dispersing in ddH_2_O. The thermal property of the prepared sample was analyzed using a Q50 thermogravimetric analyzer (TGA) from TA Instruments (New Castle, DE, USA) while using a platinum pan and then purged with nitrogen at 60 mL/min. The temperature was raised from room temperature to 700 °C at a heating rate of 10 °C/min. All of the samples were dried in a 37 °C oven for 24 h before analysis.

The element content of the prepared sample was analyzed using an inductively coupled plasma optical emission spectrometer (ICP-OES, 710-ES, Varian Inc., Palo Alto, CA, USA). All of the samples were digested in 37% HCl for 2 h at 60 °C and diluted with ddH_2_O into 15 mL before filtration through a 0.22 μm PVDF filter. The chemical structure of prepared sample was analyzed using X-ray diffraction (XRD, D2 PHASER, Bruker, MA, USA) using Cu-Kα radiation (λ = 1.54060 Å). The 2θ range was from 10 to 70° with 0.02° increment. All of the samples were dried in a 37 °C oven for 24 h before measurements. The crystalline size was calculated by the Debye–Scherrer equation.

(1) $$d=\frac{k\times \lambda }{\beta \times cos\theta }$$

In Equation (1), d is the mean size of the crystalline domains, k is a dimensionless shape factor (0.9), λ is the X-ray wavelength, β is the line broadening at half the maximum intensity, and θ is the Bragg angle. The magnetic moment of prepared sample was analyzed using a superconducting quantum interference device (SQUID) magnetometer (MPMS XL-7, Quantum Design, San Diego, CA, USA). The magnetic field-dependent magnetization was measured from −10,000 to 10,000 Oe at 298 °K. All of the samples were dried in a 37 °C oven for 24 h and 40–50 mg of samples was analyzed. The magnetic field guidance of prepared samples was tested in PBS by attaching a magnet (1700 gauss) at side of a tube containing 1 mg/mL nanoparticle for 5 min.

### 3.5. Fibrin Binding Efficiency and Fibrinolysis Asaasy

An agar solution was prepared by dissolving 0.2 g low-melting agar in 10 mL pH 7.4 Tris buffer and heated in a microwave. To induce clotting, 5 mg fibrinogen in 1 mL pH 7.4 Tris buffer, 10 mL clotting factor solution (1.8% NaCl and 0.3% CaCl_2_), and 5 mL thrombin solution (25 U) were added to the agar solution under stirring. For fibrin binding efficiency, 100 μL solution prepared above was added into a 96-well cell culture plate and incubated at 4 °C for 30 min to form solidified fibrin gel. To prepare fluorescently labelled nanoparticles, 5 μl Cy 5.5 (1 mg/mL) in dimethyl sulfoxide was added to the organic solvent mixture during PMNP and pPMNP preparation. For fibrin binding ability, 0.2 mL of test sample (PBS, 0.1 mg/mL PMNP or 0.1 mg/mL pPMNP) was added to each well and shaken for 10 min. After washing twice with PBS, microscopic images were taken using an inverted fluorescence microscope (Olympus IX71, Tokyo, Japan). 

For the agar plate fibrinolysis assay, the solution prepared above was poured into a 15 cm cell culture dish and spread evenly to obtain a homogeneous gel, which was then placed in a 4 °C refrigerator and incubated for 30 min to form solidified fibrin gel. After making nine 4-mm diameter holes on the agar plate as sample reservoirs, 10 μL of sample (20 μU rtPA) and 15 μL plasminogen prepared in PBS (1 mg/mL) were added into each hole and then incubated for 24 h at 37 °C to induce fibrinolysis. The fibrinolytic activity was determined by measuring the thickness of the clear zone around the sample hole well induced by the fibrinolytic activity of rtPA. The dose-dependent sensitivity of the test was confirmed from the same agar plate assay with 10 to 30 μU free rtPA following the same procedure.

To study the inhibition of rtPA by PAI-1, 50 μM rtPA (free rtPA, PMNP-rtPA, or pPMNP-rtPA) and 50 μM PAI-1 were mixed in 40 μL 0.1 M Tris buffer (pH 7.4). After incubation at 37 °C for different times, 20 μL Tris buffer and 20 μL S-2288 substrate (1 mM) were added and incubated for another 30 min at 37 °C, followed by adding 20 μL acetic acid (20% (*v/v*)) to stop the reaction. The amidolytic activity was calculated from the solution absorbance at 405 nm after magnetic separation using an ELISA reader, as described above.

### 3.6. Determination of Thrombolysis Using Rotational Thromboelastometry 

Thromboelastography is a method to test blood coagulation efficiency by measuring the viscoelastic properties of developing whole blood clot and used in this study for in vitro thrombolysis assays from the percentage of clot that has lysed at different time points after rtPA treatment [53]. The whole blood that was obtained from Sprague-Dawley (SD) rats using cardiac puncture was citrated and incubated at 37 °C for 30 min before use. Thrombus formation was initiated by adding 300 μL whole blood, 20 μL PBS or rtPA sample (28.9 μU/mL), and 20 μL 12 mM CaCl_2_ in the sample cup, after which a thromboelastogram could be recorded with time using rotational thromboelastometry (ROTEM, Delta2000, Tem Innovations GmbH, Munich, Germany). The clot lysis index at different time points was calculated by dividing the amplitude at a given time with the maximum amplitude corresponding to the maximum clot firmness from the thromboelastogram to evaluate the thrombolysis effect that is induced by rtPA in vitro.

### 3.7. In Vitro Biocompatibility and Hemocompatibility

To test biocompatibility, the in vitro cytotoxicity of nanoparticles was determined using NIH 3T3 cells and MTT assays. Briefly, 1 × 10^4^ NIH 3T3 cells were seeded in a 96-well cell culture plate and then cultured in DMEM medium supplemented with 10% fetal bovine serum at 37 °C in 5% CO_2_ atmosphere. After 24 h, the medium was removed and replenished with samples solution containing nanoparticles prepared in fresh medium and cultured for 24 and 48 h before measuring the mitochondria activity by the MTT assay. For MTT assay, the medium was removed and 100 μL MTT solution (10 μL 5mg/mL MTT reagent diluted with 90 μL medium) was added to each well and then incubated at 37 °C in 5% CO_2_ for 2 h. After dissolving the formazan crystals with dimethyl sulfoxide, the solution absorbance in each well was determined while using a microplate reader (Synergy HT, BioTek, Winooski, VT, USA) at 570 nm (OD_570_). 

To test blood compatibility, the possible hemolysis effect that was induced by nanoparticles was analyzed. Briefly, rat whole blood was collected, washed, and then diluted ten times with in volume with PBS first. 0.3 mL diluted red blood cell solution was mixed with 1.2 mL test sample solution and gently shaken before incubation at 37 °C for 2 h. Deionized water and PBS were used as the positive and negative controls, respectively. After centrifugation, the absorbance of the supernatant solution was recorded using a UV-Vis spectrophotometer from 500 to 650 nm. The difference in solution absorbance at 540 nm (OD_540_) between the sample and the negative control was divided by the difference in OD_540_ between the positive control and the negative control to calculate the hemolysis ratio.

### 3.8. In Vitro Blood Clot Lysis

For static clot lysis, a blood clot was prepared from rat whole blood after diluting with equal volume PBS. The diluted blood (0.5 mL) was mixed with 100 μL thrombin solution (5 U/mL) in 1.8% NaCl and 0.3% CaCl_2_ solution in a 4 mL vial and pipetting for 20 times to form the clot. After 30 min, the vial was washed twice with PBS and 50 μL of sample (25 μU rtPA activity) was added to the vial containing 2 mL PBS. The vial was placed in a 37 °C oven for 10 min before measuring absorbance of the supernatant solution at 415 nm using a UV-Vis spectrophotometer. For the sample under magnetic guidance, a magnet (80 mm × 50 mm × 10 mm, 1700 gauss) was placed at the bottom of the vial during the incubation period. 

For dynamic clot lysis, 1 mL diluted blood was mixed with 0.2 mL thrombin solution and pipetting for 20 times. The blood solution was placed in a silicone tube (4 mm diameter) and then incubated at room temperature for 1 h. After clot formation, the tube was cut into 5-mm long pieces and the blood clot was flushed out from the tube with PBS. The blood clot was placed in a 4 mL vial and 50 μL of sample (50 μU rtPA activity) was added to the vial containing 4 mL PBS. The vial was incubated in a 37 °C or 43 °C oven in a Multi Bio RS-24 programmable rotator at 10 rpm for 10 min and the absorbance of the supernatant solution at 415 nm was measured using a UV-Vis spectrophotometer.

The experiment was performed at 37 °C using a water bath for clot lysis in a flow model in a flow system employing flow occlusion induced by a blood clot (Figure 12a). A 4 mm diameter × 5 mm length blood clot prepared above was lodged tightly within a reduction point at the bottom of a tube where the inner diameter decreased from 4 mm to 3 mm. A fluid pressure gradient was generated to start flow of PBS using a syringe pump at 0.5 mL/min from the top while a magnet 80 mm × 50 mm × 10 mm, 1700 gauss) was placed below the clot for magnetic guidance. At time zero, different samples were introduced into the flow system by injecting 200 μL PBS, pPMNP, rtPA, or pPMNP-rtPA solution (100 μU rtPA) from a side opening and the reperfusion time for dissolution of the clot was measured to determine the lysis efficiency.

### 3.9. In Vivo Thrombolysis Using Rat Embolic Model

A rat embolic model was used with the protocols that the Institutional Animal Care and Use Committee of Chang Gung University approved to determine the thrombolysis efficacy in vivo. Male SD rats were anesthetized by intraperitoneal injection with Inactin^®^ (100 mg/kg). Mean arterial pressure was measured by carotid artery cannulation and a pressure transducer. The right iliac artery was cannulated with the tip of the catheter and reached to the bifurcation of abdominal aorta. A 3 mm × 3 mm blood clot was introduced from the right iliac artery, and lodged in the left iliac artery upstream of the pubic epigastric artery. The sample (pPMNP, rtPA, and pPMNP-rtPA) was administered via the right iliac artery and guided by a 0.5 T magnet from the abdominal aorta and iliac artery branch to the iliac artery and the femoral artery for 1 min, followed by magnetic guiding to the lower extremities for 4 min. The guiding cycle was repeated four times. The rate of iliac blood flow (IBF) and aortic blood flow (ABF) were determined by placing ultrasonic flow probes (T206, Transonic System Inc., Ithaca, NY, USA) on the left iliac artery and the aorta upstream of left renal artery, respectively. Real-time, high-resolution blood flow images at different time points after treatment were carried out by laser speckle contrast analysis while using a MoorFLPI laser speckle contrast imager from Moor Instruments (Devon, UK).

### 3.10. Statistical Analysis

All of the results were presented as mean ± standard deviation (SD), except for in vivo experiments, which were presented as mean ± standard error of mean (SEM). For statistical analysis, the data are subject to one-way analysis of variance (ANOVA) and Tukey’s test with statistical significance declared with a *p* value of less than 0.05.

## 4. Conclusions

In the study, we demonstrated the preparation of functional PLGA MNP modified through avidin-biotin interaction for the immobilization of fibrin-avid peptide (GPRPPGGSKGC) and thrombolytic drug (rtPA) in dual targeted thrombolytic therapy in vitro and in vivo. Iron oxide magnetic nanoparticles were well encapsulated in PLGA MNP, as confirmed by physico-chemical characterization, which allowed for drug delivery through magnetic guidance. Under the optimized preparation conditions, rtPA retains the same clot-lysis efficiency after conjugation to the dual targeted nanoparticle (pPMNP-rtPA) to facilitate its use in thrombolytic therapy. The magnetic guiding and fibrin binding ability associated with the dual targeted nanoparticle further reveal the preference of pPMNP-rtPA over free rtPA for in vitro and in vivo thrombolysis. The dual targeting thrombolytic nano-drug will have the potential to improve the efficacy of a thrombolytic drug and facilitate in vivo thrombolysis for future clinical application.

## Figures and Tables

**Table 1:** The average particle size, polydispersity index (PDI), zeta potential, and crystal size of different nanoparticles.

Sample ^1^	Average Diameter ^2^ (nm)	PDI	Zeta Potential (mV)	Crystal Size ^3^ (nm)
MNP	230.7 ± 17.1	0.19 ± 0.05	12.1 ± 2.9	10.2
OMNP	229.3 ± 18.5	0.20 ± 0.01	−17.6 ± 0.9	10.5
PMNP	252.7 ± 13.4	0.19 ± 0.04	−30.0 ± 1.3	10.3
PMNP-avidin	278.8 ± 19.1	0.21 ± 0.02	−25.8 ± 0.4	11.7
PMNP-rtPA	291.2 ± 27.3	0.21 ± 0.04	−24.4 ± 1.1	10.7
pPMNP-rtPA	321.1 ± 26.9	0.22 ± 0.05	−22.1 ± 2.0	10.9

^1^ MNP: iron oxide magnetic nanoparticles, OMNP: oleic acid coated magnetic nanoparticles, PMNP: PLGA magnetic nanoparticles, PMNP-avidin: avidin-conjugated PLGA magnetic nanoparticles, PMNP-rtPA: rtPA-conjugated PLGA magnetic nanoparticles, pPMNP-rtPA: peptide/rtPA-conjugated PLGA magnetic nanoparticles. ^2^ Determined from dynamic light scattering (DLS). ^3^ Determined from x-ray diffraction (XRD).

**Table 2:** The residual weight at 700 °C from thermogravimetric analysis (TGA) and the Fe_3_O_4_ content determined from inductively coupled plasma-optical emission spectrometry (ICP-OES) and superconducting quantum interference device (SQUID).

Sample ^1^	Residual Weight from TGA (%)	Fe_3_O_4_ from ICP-OES (%)	Fe_3_O_4_ from SQUID (%)
MNP	97.9	97.8 ± 1.6	100.0 ± 0.05
OMNP	87.7	87.1 ± 2.1	90.9 ± 0.11
PMNP	14.0	13.5 ± 0.3	15.5 ± 0.06
PMNP-avidin	13.4	12.3 ± 0.8	14.4± 0.11
PMNP-rtPA	17.1	10.5 ± 0.3	13.3 ± 0.07
pPMNP-rtPA	16.9	10.1 ± 0.4	13.5 ± 0.07

^1^ MNP: iron oxide magnetic nanoparticles, OMNP: oleic acid coated magnetic nanoparticles, PMNP: PLGA magnetic nanoparticles, PMNP-avidin: avidin-conjugated PLGA magnetic nanoparticles, PMNP-rtPA: rtPA-conjugated PLGA magnetic nanoparticles, pPMNP-rtPA: peptide/rtPA-conjugated PLGA magnetic nanoparticles.
